# Data-driven analysis of heterogeneous gait subgroups and ground reaction forces based on integrated center of pressure–center of mass dynamics in poststroke hemiparesis

**DOI:** 10.1371/journal.pone.0354290

**Published:** 2026-07-20

**Authors:** Kimihiko Mori, Tatsuya Teramae, Masanori Wakida, Naoto Mano, Yuta Chujo, Takayuki Kuwabara, Meguru Taguchi, Kimitaka Hase, Tomoyuki Noda

**Affiliations:** 1 Faculty of Rehabilitation, Kansai Medical University, Hirakata, Osaka, Japan; 2 Department of Brain Robot Interface, Computational Neuroscience Laboratories, Advanced Telecommunications Research Institute International, Seika, Kyoto, Japan; 3 Department of Rehabilitation Medicine, Kansai Medical University Hospital, Hirakata, Osaka, Japan; 4 Department of Rehabilitation Medicine, Kansai Medical University Kori Hospital, Neyagawa, Osaka, Japan; 5 Department of Rehabilitation Medicine, Kansai Medical University, Hirakata, Osaka, Japan; Pennsylvania State University Main Campus: The Pennsylvania State University - University Park Campus, UNITED STATES OF AMERICA

## Abstract

**Introduction:**

Hemiparetic gait is characterized by abnormal ground reaction forces (GRFs) with impaired control of the center of mass (CoM) relative to the center of pressure (CoP). Although both anteroposterior and mediolateral gait control have been examined separately, how their integrated stance-phase–derived CoP–CoM interactions and local CoP-based features relate to GRF characteristics remains unclear.

**Objective:**

This exploratory study aimed to explore the relationships between CoP–CoM and CoP-based dynamics and GRFs and descriptively identify candidate gait subgroups of individuals with poststroke hemiparesis using a data-driven clustering approach.

**Methods:**

Seventy-eight community-dwelling individuals with poststroke hemiparesis participated in a three-dimensional gait analysis. Stance-phase–derived CoP–CoM parameters of the transverse plane and local CoP-based loading metrics were extracted during the paretic stance phase. Relationships between these gait metrics and GRFs, particularly early braking force, propulsion, and late braking force, were examined using nonparametric correlation analyses. K-means clustering was performed to explore candidate gait subgroups, and inter-cluster differences were exploratorily examined.

**Results:**

Across all participants, several CoP–CoM interaction metrics showed significant correlations with GRFs. In particular, insufficient forward progression of the CoM relative to the CoP during late stance showed a strong correlation with late braking force (r_s_ = 0.79). Clustering suggested the presence of four candidate gait subgroups characterized by differing combinations of anteroposterior and mediolateral CoP–CoM dynamics and local CoP loading features. However, the results of clusters with small sample sizes warrant cautious interpretation.

**Conclusions:**

Integrated stance-phase–derived CoP–CoM dynamics and CoP-based parameters may highlight heterogeneity in hemiparetic gait and may have meaningful associations with GRF characteristics. The candidate gait subgroups should be interpreted as hypothesis-generating and may offer a descriptive framework for understanding diverse gait disorders.

## Introduction

Hemiparetic gait after stroke is characterized by lower limb motor dysfunction and accompanied by heterogeneous impairments across individuals [1,2], including abnormal gait patterns [1], reduced walking speed [3], and impaired symmetry of ground reaction force (GRF) [4] and spatiotemporal parameters [5,6], resulting in limited walking efficiency and activities of daily living [7]. The GRF imparts acceleration to the center of mass (CoM) in coupled anteroposterior–mediolateral (AP–ML) directions; therefore, paretic limb control of the CoM should be understood as an integrated AP–ML interaction mediated by the GRF. Regarding AP control, the leading limb angle during early stance and the trailing limb angle, as defined by the relative position of the CoM to the center of pressure (CoP) during late stance, are related to the AP component of the GRF; however, the former has been related to early braking force (EBF) [8] and the latter has been related to propulsive force (PF) [9]. Regarding ML control, the foot progression angle (FPA) [3] and lateral foot placement relative to the CoM are associated with walking speed and stability [10,11], and cane use is considered compensation for ML instability [12]. Furthermore, CoP–CoM control in the transverse plane plays a crucial role in stabilizing gait and adapting to neurological deficits [13,14]. Therefore, both AP and ML controls should be understood as a coupled factor rather than as isolated factors.

While the global CoP–CoM relationship captures overall balance and propulsion strategies, local features of the CoP trajectory on the paretic side provide additional insight into the ankle control mechanisms that underlie these dynamics. In particular, difficulty with hindfoot loading during stance and a concomitant reduction in anterior displacement of the CoP are associated with slower walking speed [15] and the occurrence of late braking force (LBF) [16], with lateral CoP deviation further necessitating cane use [12]. Data of healthy individuals and individuals with subacute stroke have shown that AP and ML shifts in the CoP are linked to ankle inversion control and eversion control as well as trunk control [17,18], suggesting the need to integrate local CoP trajectories arising from ankle control with global CoP–CoM dynamics to capture the dynamic characteristics of hemiparetic gait. Moreover, characteristics of hemiparetic gait should be interpreted across the stance phase rather than at a single point, as events at one phase can influence those at subsequent phases; excessive paretic braking forces during early stance often hinder forward CoM progression and reduce backward foot placement during late stance [19], highlighting the interdependence of early and late stance dynamics. Nevertheless, how stance-phase–derived features of AP–ML interactions, which integrate the global trajectory of the CoP–CoM with the local CoP trajectory and loading in the transverse plane, are associated with AP components of the GRF, particularly PF and LBF, is unclear. This gap is clinically important and particularly relevant given that these integrated AP–ML CoP–CoM interactions are likely to vary across individuals depending on their walking ability [19] and compensatory strategies related to the GRFs [16,20].

Therefore, combined stance-phase–derived CoP–CoM and CoP-based parameters may help capture the heterogeneous biomechanical characteristics of hemiparetic gait across individuals. However, because each parameter carries distinct clinical relevance, it remains unclear which combination of parameters most meaningfully relates to GRF characteristics across individuals. Data-driven clustering offers a systematic approach to address this question, and has been increasingly applied to characterize the heterogeneity of post-stroke gait. Prior studies using spatiotemporal, kinematic [21,22], and limb kinematic variables [23] have identified biomechanically distinct subgroups associated with differences in motor impairment severity, compensatory strategies, and walking ability. However, as highlighted in a recent narrative review [24], cluster differentiation has often been dominated by walking speed, and approaches integrating AP–ML CoP–CoM dynamics with GRF-related kinetic features remain unexplored. Exploring candidate gait subgroups based on these integrated dynamics could advance understanding of the diverse GRF characteristics in poststroke hemiparesis and inform targeted rehabilitation strategies.

This study aimed to characterize the heterogeneous biomechanical characteristics of hemiparetic gait by extracting stance-phase–derived CoP–CoM and CoP-based parameters, examining their associations with GRF components from an integrated AP–ML perspective, and applying data-driven clustering to explore candidate gait subgroups. Based on the biomechanical relationships, we hypothesized that clustering would reveal candidate subgroups characterized by differing AP–ML CoP–CoM control patterns, in which insufficient anterior CoP displacement combined with lateral foot placement compensation would contribute to reduced PF and increased EBF, while lateral CoP deviation and decreased local anterior CoP displacement would be associated with increased LBF [16].

## Methods

### Participants

We performed a retrospective analysis of data from a database at our institution. This study included 78 community-dwelling individuals with poststroke hemiparesis who were capable of walking independently on a level surface with or without the use of a T-handle cane. The exclusion criteria were the inability to walk independently without assistance from a therapist and the presence of bilateral lesions, lower extremity joint pain, or other neurological or musculoskeletal disorders that could affect walking. We measured Fugl–Meyer Assessment of Lower Extremity synergy (item E, Stages II–IV; maximum score = 22) [25] and sensory scores (item H; lower extremity items only; maximum score = 12) and assessed the daily use of canes among individuals after stroke. Data were collected at three university hospitals as part of routine medical care or other studies between March 1, 2018 and March 1, 2024. This study was conducted in accordance with the Declaration of Helsinki, and the protocol was approved by the University Human Research Ethics Committee (Kansai Medical University, no. 2022150). Data were first accessed for research purposes on April 1, 2024. Authors did not have access to information that could directly identify individual participants during or after data collection, and all data were anonymized prior to analysis. Informed consent was obtained through an opt-out process.

### Gait analysis

Gait performance was evaluated using an optical three-dimensional motion capture system (Locus 3D MA-3000; Anima Corp., Tokyo, Japan) and two force plates (MG-1190; Anima Corp.). The system comprised 12 infrared cameras and 1.2-m force plates with sampling rates of 100 Hz and 1000 Hz, respectively, that were synchronized. The 22 reflective markers had a diameter of 12 mm and were placed on the body surface of the acromion, anterosuperior iliac spine, posterosuperior iliac spine, greater trochanter, medial and lateral femoral epicondyles, medial and lateral malleolus, first metatarsal head, fifth metatarsal head, and heel, bilaterally.

GRF data were collected as participants walked at a self-selected speed along a 6-m walkway. To avoid unnatural gait adaptations, participants were instructed to walk naturally without attempting to adjust their steps to target the force plates. The use of a specialized cane (MK-1000A; Anima Corp.) that enables separation of GRF components from the cane and foot when both contact the same force plate was permitted to the minimum extent necessary to prevent falls caused by lateral instability during walking, particularly for individuals with lower walking ability. However, all data used in the present study were measured under the condition without an ankle–foot orthosis.

**Fig 1:**
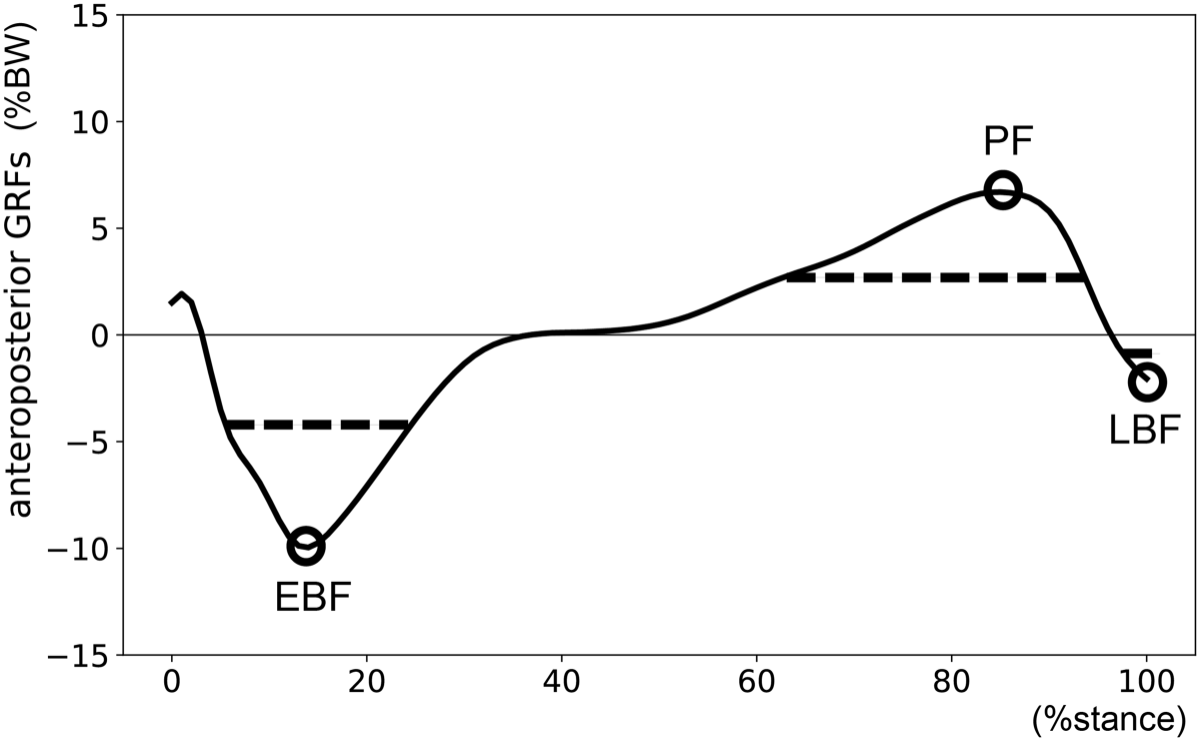
Definition of anteroposterior (AP) ground reaction forces (GRFs) during the stance phase. Early braking force (EBF) was defined as the negative AP GRF phase following the heel strike transient until the onset of the propulsive phase. Propulsive force (PF) was defined as the subsequent positive AP GRF phase until the transition to negative values or foot-off. Late braking force (LBF) was defined as the negative AP GRF phase following the propulsive phase until foot-off. Peak values are indicated by circles. Mean values of each phase are shown as dashed lines. Positive values represent the anterior component and negative values indicate the posterior component.

Marker trajectories and GRFs were filtered using second-order Butterworth low-pass filters at 10 Hz and 20 Hz, respectively. Gait events were identified based on the vertical GRF thresholds as described previously [26]. Specifically, initial contact was defined as the time when the vertical GRF exceeded 5% of body weight (BW), and foot-off was defined as the point when it decreased below 5% BW. The stance phase from the paretic initial contact to foot-off was normalized from 0% to 100%. The whole-body CoM and paretic CoP were calculated using marker locations and force plate signals, respectively. The CoM was estimated using a simplified seven-segment rigid body model including the bilateral feet, shanks, thighs, and trunk, as described by Yamaguchi et al. [27]. The results of at least three paretic stance trials of all individuals except one were averaged; for that one individual, the results of two paretic stance trials were averaged. Walking speed and stance duration relative to the gait cycle were calculated using at least four strides. The AP GRFs, such as the peak and mean values of the EBF during early stance, PF during late stance, and subsequent LBF, were calculated and normalized according to BW (%BW) (Fig 1).

### Data processing

**Fig 2:**
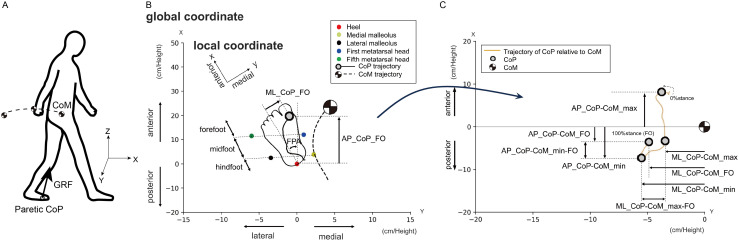
Trajectory of the center of mass (CoM) and center of pressure (CoP) and determinants of stance-phase–derived gait parameters. **(A)** Schematic illustration of the relative position of the CoM and CoP during gait and the ground reaction force (GRF). **(B)** Trajectories of the CoM and CoP in the global and local coordinate systems in the transverse plane. In the local coordinate system, the foot is divided into three anatomical regions (hindfoot, midfoot, and forefoot), which correspond to the regions used to define hindfoot_CoP_duration, midfoot_CoP_duration, and forefoot_CoP_duration (see Table 1 for definitions). The anteroposterior CoP position at foot-off (AP_CoP_FO), the mediolateral CoP position at foot-off (ML_CoP_FO), and the foot progression angle (FPA) are also indicated. **(C)** Parameters derived from the trajectory of the CoP relative to the CoM in the transverse plane, including anteroposterior parameters (AP_CoP–CoM_max, AP_CoP–CoM_min, AP_CoP–CoM_FO, and AP_CoP–CoM_min-FO) and mediolateral parameters (ML_CoP–CoM_max, ML_CoP–CoM_max-FO, ML_CoP–CoM_FO, and ML_CoP–CoM_min). All parameters are defined in Table 1.

To ensure consistency during the analysis, the ML coordinates of the markers and CoP were standardized to account for the side of hemiparesis. Thus, the X-axis and Y-axis in the global coordinate system were defined as the anterior and medial directions, respectively, with positive values corresponding to the paretic limb; however, in the local coordinate system, the X-axis and Y-axis represented the anterior and medial directions, respectively, which were defined based on the foot (Fig 2A and 2B). In the local coordinate system, the X-axis was defined as the line extending from the midpoint between the first and fifth metatarsal head markers to the heel marker. The Y-axis (medial direction) was defined as the perpendicular direction to the X-axis in the transverse plane. The origin of this coordinate system was set at the heel marker. This framework was applied to all relevant markers and CoP measurements to allow direct comparisons across participants.

**Table 1:** Definitions and clinical significance of gait metrics.

	Gait metrics	Definitions	Clinical significance
**Global coordinate**	**AP_CoP–CoM_max**	**Maximal anteroposterior distance of CoP relative to CoM**	**Leading limb angle (forward foot placement)**
	**AP_CoP–CoM_min**	**Minimal anteroposterior distance of CoP relative to CoM**	**Trailing limb angle (backward foot placement)**
	**AP_CoP–CoM_FO**	**Anteroposterior distance of CoP relative to CoM at foot-off**	**Trailing limb angle (backward foot placement)**
	**AP_CoP–CoM_min-FO**	**Difference between “AP_CoP–CoM_min” and “AP_CoP–CoM_FO”**	**Insufficient forward progression of the CoM** **relative to the anterior shift of the CoP**
	**ML_CoP–CoM_max**	**Maximal medial distance between CoP and CoM**	**Lateral weight shift during midstance**
	**ML_CoP–CoM_max-FO**	**Changes in lateral distance between CoP and CoM**	**Lateral balance from midstance to foot-off**
	**ML_CoP–CoM_FO**	**Mediolateral distance of CoP relative to CoM at foot-off**	**Lateral balance at foot-off**
	**ML_CoP–CoM_min**	**Minimal mediolateral distance of CoP relative to CoM**	**Lateral balance during stance**
	**AP_CoP_FO**	**Anterior position of CoP at foot-off**	**Toe catching caused by premature swing initiation**
	**FPA**	**Foot progression angle**	**Lateral control by foot rotation**
**Local coordinate**	**ML_CoP_FO**	**Mediolateral position of CoP at foot-off**	**Ankle eversion control**
	**Forefoot_CoP_duration**	**Proportion of loading time on a forefoot relative to stance time**	**Loading function on a forefoot**
	**Midfoot_CoP_duration**	**Proportion of loading time on a midfoot relative to stance time**	**Loading function on a midfoot**
	**Hindfoot_CoP_duration**	**Proportion of loading time on a hindfoot relative to stance time**	**Loading function on a hindfoot**

Gray font indicates gait metrics that were excluded from clustering because of their high correlation with other metrics (|r| > 0.80).

AP, anteroposterior; BW, body weight; CoM, center of mass; CoP, center of pressure; FO, foot-off; FPA, foot progression angle; max, maximal; min, minimum; ML, mediolateral.

Data processing involved calculating the positions of key markers, trajectories of the CoP, and coordinates of the CoM in the transverse plane during the stance phase, followed by calculating relative values to compare measurements across participants. Key foot markers, including the markers of the heel, lateral malleolus, medial malleolus, fifth metatarsal head, and first metatarsal head, were analyzed. Coordinates of these markers were adjusted relative to the initial heel position and normalized according to the height of the participants. The CoP and CoM trajectories were adjusted using the same normalization method.

The definitions and clinical significance of all 14 gait metrics are summarized in Table 1, and their derivation from the CoP and CoM trajectories is illustrated in Fig 2. The horizontally projected positions of the CoP relative to the CoM in the global coordinate system in the AP (X-axis) and ML (Y-axis) directions were calculated to understand their dynamic relationship during the stance phase (Fig 2C). AP_CoP–CoM_min and AP_CoP–CoM_max capture the posterior and anterior displacement of the CoP relative to the CoM—constructs conceptually analogous to the trailing and leading limb angles [8,9]—and the ML CoP–CoM parameters characterize mediolateral divergence between the CoP and CoM, related to lateral foot placement and pelvis displacement [11]. These relative values were further used to derive specific metrics including AP_CoP–CoM_min-FO, which quantifies the anterior shift of the CoP relative to the CoM from its most posterior point to foot-off and is a novel parameter not previously reported. Additionally, at the end of the stance phase, the anterior position of the CoP along the X-axis relative to the initial heel position in the global coordinate system (AP_CoP_FO) is a newly defined parameter that characterizes the excessive anterior shift of CoP during preswing, which may reflect toe catching caused by premature swing initiation before sufficient unloading of the limb [20,28]. The direction of foot progression was determined by calculating the angle between the central axis of the foot (from the midpoint between the first and fifth metatarsal heads to the heel) and the X-axis, with angles > 0° indicating a toe-out position, consistent with the role of foot yaw in lateral stability [29].

In contrast, for gait characteristics in the local coordinate system, the foot was divided into the following three regions: hindfoot (heel to malleoli); midfoot (malleoli to metatarsal heads); and forefoot (anterior to metatarsal heads). The percentage of the stance time during which the load was applied to each region was calculated as discrete parameters of CoP progression from hindfoot to forefoot, a feature previously associated with walking ability in hemiparetic gait [15]. To quantify ankle inversion or eversion control at foot-off (ML_CoP_FO), the ML position of the CoP relative to the central axis of the foot (Y-axis) was determined by calculating the perpendicular intersection from the heel to the line connecting the first and fifth metatarsal heads, reflecting a construct related to ankle muscle control of the mediolateral CoP position during stance [17].

### Statistical analysis

To reduce redundancy among gait parameters, we applied a step-by-step variable selection process based on pairwise Pearson correlation coefficients. In each functional category, when two or more variables showed high correlation (|r| > 0.80, more conservative than the r > 0.9 criterion used in a comparable clustering study [30]), the variable with a lower correlation coefficient relative to the others was retained. This process was conducted independently for each feature category (e.g., ML CoP–CoM metrics, AP CoP–CoM metrics, and CoP duration metrics). The final set of variables, which included only those with minimal redundancy, was defined prior to clustering and subsequently used for the clustering analysis (Table 1).

The remaining features were standardized using z-score normalization prior to clustering. Then, a K-means clustering analysis was performed to explore candidate gait subgroups among participants. The optimal number of clusters was primarily determined based on the silhouette coefficient [31] and the elbow method was used as a supplementary tool. To address initialization dependency, k-means clustering was performed using k-means++ seeding with 1,000 independent random initializations, selecting the solution with the lowest inertia as the reference. Cluster stability was further assessed by computing the Adjusted Rand Index (ARI) [32] between the reference solution and 100 repeated runs for k = 3, 4, and 5. The candidate clusters were additionally visualized by projecting the clustering features onto the first two principal components to examine cluster separation in the feature space.

To explore the relative contribution of each metric used in the clustering analysis, we applied the mean decrease accuracy method using the randomForest package in R, which is similar to the method described by Kettlety et al. [31]. The importance scores were normalized to the sum of 100%, thus making it easier to compare the relative influence of each metric on the clustering model. Because LBF is defined as the negative AP GRF phase following the propulsive phase, participants in whom no clear propulsive phase was identified were excluded from all LBF-related analyses. Correlations between GRF data and gait characteristics across all participants were exploratorily analyzed. Data normality was assessed using the Shapiro–Wilk test for all variables included in the correlation analyses. Because GRFs metrics were not normally distributed, Spearman’s rank correlation was used throughout.

Differences in GRF data and gait characteristics among the clusters were determined after the clustering analysis results were analyzed. Because cluster C comprised only four participants, the Kruskal–Wallis rank-sum test was used to determine the presence of significant differences in the variables across groups. To quantify the effect size, the epsilon-squared value was calculated as a measure of association strength. Multiple comparisons among clusters were performed using post hoc analyses with the Steel–Dwass test to determine which clusters significantly differed from each other. All analyses were performed using Python 3.10.12 and R software version 4.4.1.

## Results

Seven individuals without PF data (two in cluster B, one in cluster C, and four in cluster D) were excluded from the LBF analysis. A step-by-step variable selection process was conducted to reduce multicollinearity among the 14 predefined gait characteristic indices. As a result, the following variables were excluded because of high correlation (|r| > 0.80) with other metrics: ML_CoP–CoM_min; ML_CoP–CoM_FO (correlated with ML_CoP–CoM_max); AP_CoP–CoM_FO (correlated with AP_CoP–CoM_min); and midfoot_CoP_duration (correlated with forefoot_CoP_duration). Consequently, 10 gait characteristics were used for clustering analyses (Table 1).

### Relationship between GRFs and characteristics of hemiparetic gait

Peak and mean EBFs showed the highest correlation with AP_CoP–CoM_max (r_s_ = –0.75 and r_s_ = –0.77). Peak and mean PFs had the strongest correlation with AP_CoP–CoM_min (r_s_ = –0.91 and r_s_ = –0.91) and were significantly correlated with ML_CoP_FO, ML_CoP–CoM_max-FO, and hindfoot_CoP_duration. Peak and mean LBFs were strongly correlated with AP_CoP–CoM_min-FO (r_s_ = 0.79 and r_s_ = 0.78) and AP_CoP_FO (r_s_ = –0.58 and r_s_ = –0.60). Correlations between the other metrics and GRFs are presented in S1 Fig.

### Differences in gait characteristics among clusters

**Fig 3:**
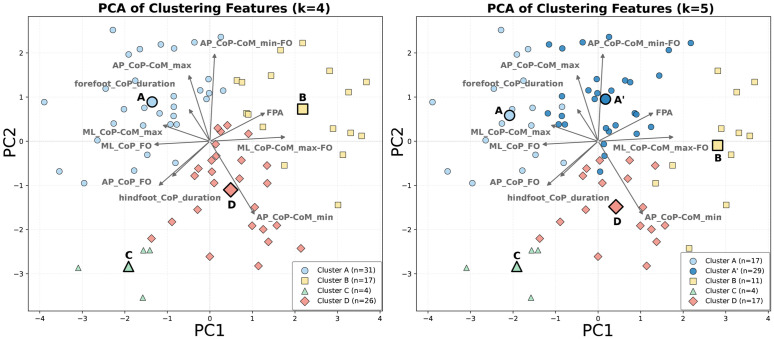
Principal component analysis of the 10 clustering features for k = 4 (A) and k = 5 (B) solutions. Each point represents one participant, and shapes and colors denote cluster membership. Large symbols indicate cluster centroids. Arrows indicate loading vectors for each of the 10 clustering variables, showing their contributions to PC1 and PC2. The first principal component (PC1; 29.6% variance explained) reflects mediolateral control variables, and the second principal component (PC2; 20.9%) reflects anteroposterior control variables. Cluster C showed complete membership overlap across both solutions.

**Fig 4:**
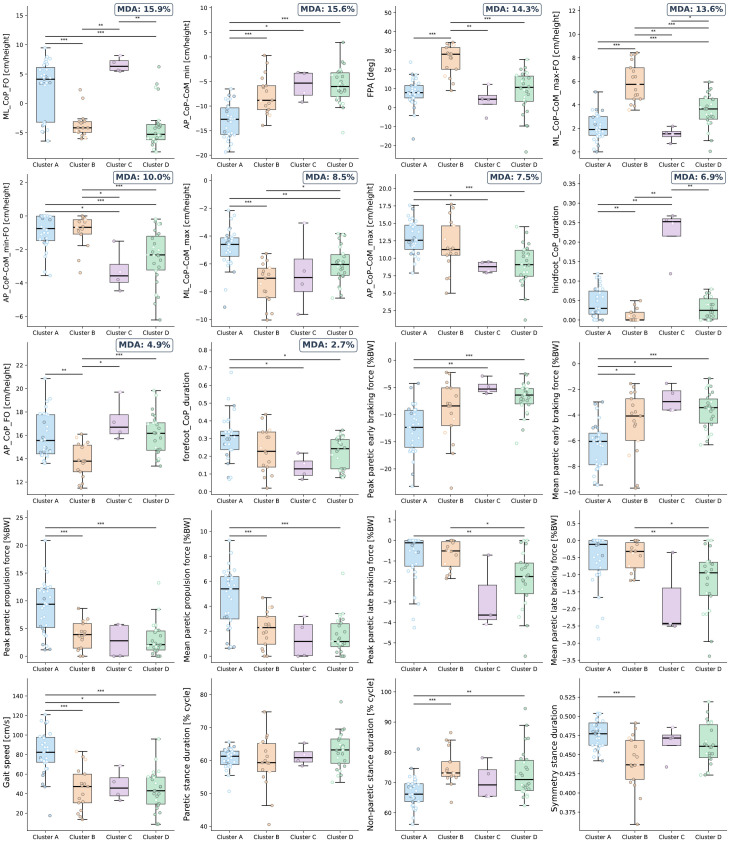
Stance-phase–derived gait parameters and ground reaction force components across the four clusters. Box plots show the distribution of 10 clustering parameters (in order of mean decrease accuracy [MDA]), ground reaction force (GRF) components, and walking and stance parameters for each cluster (Cluster A, n = 31; Cluster B, n = 17; Cluster C, n = 4; Cluster D, n = 26). Individual data points are shown as circles with jitter. Horizontal bars indicate significant pairwise differences identified by the Steel–Dwass post hoc test (* p < 0.05, ** p < 0.01, *** p < 0.001). MDA values (%) indicate the relative contribution of each parameter to cluster differentiation. Full numerical summaries (median and interquartile range) are provided in the S1 Table. Filled circles indicate participants who used a cane during gait analysis; open circles indicate those who did not. AP, anteroposterior; ML, mediolateral; CoM, center of mass; CoP, center of pressure; FO, foot-off; FPA, foot progression angle; BW, body weight.

The clustering results assigned 31, 17, 4, and 26 participants in clusters A, B, C, and D, respectively (Table 2). The silhouette coefficients were 0.163, 0.178, and 0.173 for k = 3, 4, and 5, respectively, with k = 4 yielding the highest value (S2A Fig). The elbow plot showed a gradual and continuous decline in the sum of squared errors without a distinct inflection point (S2B Fig). Mean ARI values were 0.842 (SD = 0.108), 0.723 (SD = 0.142), and 0.778 (SD = 0.129) for k = 3, 4, and 5, respectively, indicating moderate-to-good stability across initializations (S2C Fig). PCA-based visualization suggested meaningful separation in both the k = 4 and k = 5 solutions (Fig 3), supporting the selection of k = 4 based on the highest silhouette coefficient (S2A Fig). The k = 5 solution subdivided Cluster A into two subgroups, whereas Cluster C showed complete membership overlap across k = 4 and k = 5, further supporting its consistency as a candidate gait subgroup (S2D Fig). The distribution of 10 clustering parameters, including their mean decrease accuracy, and GRF components across clusters are shown in Fig 4, with full numerical summaries provided in the S1 Table for k = 4, S2 Table for k = 5.

**Table 2:** Demographic characteristics among clusters.

Characteristics	Cluster A n = 31	Cluster B n = 17	Cluster C n = 4	Cluster D n = 26	*P* (ε²)
Age	63.0 ± 13.4	61.0 ± 14.5	63.8 ± 3.3	69.5 ± 8.5	0.126 (0.04)
Height	163.4 ± 8.1	168.4 ± 7.7	164.0 ± 8.2	159.5 ± 9.6 †	0.015 (0.10)
Weight	59.1 ± 9.4	70.1 ± 12.3 *	60.5 ± 4.6	61.2 ± 11.0	0.025 (0.09)
Sex (F/M)	14/17	1/16	3/1	12/14	
Paretic side (L/R)	14/17	9/8	2/2	13/13	
Disease (hemorrhage/infarction)	15/16	8/8 [1]	2/1 [1]	14/10 [2]	
Months from onset	43.7 ± 52.0 [1]	41.5 ± 53.1 [2]	9.3 ± 1.2 [1]	72.1 ± 73.7 [4]	0.28 (0.01)
Fugl-Meyer Assessment synergy score	18.3 ± 3.9 [4]	15.4 ± 3.3 [2] *	20.8 ± 1.0 †	18.0 ± 3.7 [6]	0.011 (0.13)
Fugl-Meyer Assessment sensory score	10.7 ± 1.9 [2]	10.0 ± 2.4 [2]	10.8 ± 2.5	10.5 ± 2.8 [4]	0.79 (0)
Daily use of walking aids (cane)	7 (24%) [2]	12 (80%) [2] ‡	3 (75%)	17 (74%) [3] ‡	
Walking aids (cane) used during measurements	8 (26%)	15 (88%) ‡	3 (75%)	18 (69%) ‡	

Missing data points are shown as [n].

*P* values were obtained from the Kruskal–Wallis test, and effect sizes were calculated using epsilon-squared (ε²).

Pairwise comparisons between clusters were conducted using the Steel–Dwass test to identify significant differences.

*Significant difference compared with cluster A (*p* < 0.05)

† Significant difference compared with cluster B (*p* < 0.05)

A multiple pairwise Fisher’s exact test with the Bonferroni correction was performed for sex, paretic side, disease, and walking aid use.

‡ Significant difference compared with cluster A (corrected *p* < 0.05/6)

F, female; L, left; M, male; R, right

Cluster B showed significantly greater ML_CoP–CoM_max and ML_CoP–CoM_max-FO than cluster A, indicating greater lateral deviation of the CoP relative to the CoM and a larger lateral shift toward foot-off. AP_CoP–CoM_min was also significantly reduced, indicating less posterior displacement of the CoP relative to the CoM. In terms of foot control and CoP-based parameters, cluster B exhibited a significantly larger FPA, smaller hindfoot_CoP_duration, and smaller AP_CoP_FO and laterally shifted ML_CoP_FO compared with cluster A.

In cluster C, AP_CoP–CoM_max and AP_CoP–CoM_min were significantly smaller than in cluster A, indicating reduced anterior and posterior displacement of the CoP relative to the CoM, and AP_CoP–CoM_min-FO was significantly larger. Regarding CoP-based parameters, hindfoot_CoP_duration was significantly larger, and forefoot_CoP_duration was significantly smaller than in cluster A.

Cluster D exhibited significantly increased ML_CoP–CoM_max compared with cluster A, indicating greater lateral deviation of the CoP relative to the CoM during midstance, though significantly less than that in cluster B. Regarding CoP-based parameters, cluster D exhibited a smaller forefoot_CoP_duration and a laterally shifted CoP at foot-off (ML_CoP_FO) compared with cluster A.

Compared with clusters B and D, cluster A exhibited significantly greater peak and mean PFs (Fig 4 and visually illustrated mean AP GRF waveforms in S3 Fig). In contrast, cluster A exhibited significantly lower EBF than the other clusters, except for peak EBF in cluster B. LBFs in cluster D were significantly greater than those in clusters A and B. Additionally, walking speed in cluster A was significantly higher than that in the other clusters.

## Discussion

This study characterized the biomechanical heterogeneity of hemiparetic gait using stance-phase–derived CoP–CoM and CoP-based parameters and explored four candidate gait subgroups with differing AP–ML control patterns (Fig 4). Many of the exploratorily selected parameters showed significant associations with GRF components across all participants (S1 Fig), and the most discriminative variables further contributed to characterizing the clinically relevant gait features of each candidate subgroup, as reflected in both GRF components and walking speed.

### Relationship between the dynamics of CoM, CoP, and GRFs across all participants

In the present study, multiple global CoP–CoM relationship parameters as well as local CoP-based parameters were significantly correlated with GRFs (S1 Fig). These findings highlight the biomechanical interaction between CoM and CoP dynamics and their contributions to the generation of EBF, PF, and LBF in hemiparetic gait.

Our new parameter, AP_CoP–CoM_min-FO, was described as the reversed CoP–CoM trajectory just before foot-off and quantified as the anterior shift of the CoP from its most posterior point relative to the CoM. A larger negative value of this parameter indicated that the CoP had progressed forward more than the CoM, suggesting the excessive anterior shift of the CoP and insufficient forward progression of the CoM during late stance. An excessive anterior shift of the CoP may indicate toe catching caused by premature swing initiation during late stance [20,28], as characterized by a larger AP_CoP_FO. Insufficient forward progression of the CoM may represent halted anterior tilt of the shank during late stance, as often observed in hemiparetic gait. Importantly, AP_CoP–CoM_min-FO showed the strongest correlation with the LBF, suggesting its potential relevance in capturing insufficient forward progression of the CoM relative to the anterior shift of the CoP.

Furthermore, a correlation between AP_CoP–CoM_min-FO and AP_CoP–CoM_min (r_s_ = –0.47) was also observed, which indicated a relationship between decreased PF and increased LBF. AP_CoP–CoM_min in the transverse plane showed the highest correlation with PF, consistent with the findings of previous studies that reported that the trailing limb angle based on the CoP and pelvic CoM is strongly correlated with peak propulsion force [9]. This finding suggests that the horizontal distance between the CoP and CoM calculated in this study contributed to the anterior component of the GRF vector. However, we revealed that gait subgroups with insufficient forward progression of the CoM (lower PF) may lead to excessive anterior shift of the CoP and generate greater LBF captured by the AP_CoP–CoM_min-FO. Therefore, the reversed trajectory of CoP relative to the CoM may serve as a novel indicator of late stance dynamics, which may help characterize specific hemiparetic gait.

Additionally, ML_CoP–CoM_max-FO was correlated with PF. ML_CoP–CoM_max-FO was also correlated with ML_CoP–CoM_max, FPA, and ML_CoP_FO in terms of lateral control, suggesting that a greater lateral shift of the CoP relative to the CoM (ML_CoP–CoM_max-FO) with a toe-out posture in the global coordinate system was associated with lateral foot loading (ML_CoP_FO) in the local coordinate system (r_s_ = –0.55). This supports the idea that compensatory postural control may involve increased toe-out and lateral foot placement [29], in response to increased lateral body sway in hemiparetic gait [33]. During normal gait, when the foot is positioned laterally relative to the CoM, a compensatory control mechanism shifts the CoP medially to maintain balance [17]. However, in individuals with hemiparesis, the ability to medially shift the CoP in response to lateral foot placement could be compromised because of more severe lower limb dysfunction, as observed in cluster B. Therefore, altered lateral balance would limit PF generation after reduced posterior foot placement [10].

AP_CoP–CoM_max showed the highest correlation with EBF, consistent with the findings of previous studies that reported that the leading limb angle is associated with the posterior component of the GRF vector [8].

### Gait characteristics among clusters

The clustering analysis suggested four candidate gait subgroups based on the differing CoM and CoP dynamics. The variables with the highest mean decrease accuracy were ML_CoP_FO, AP_CoP–CoM_min, FPA, ML_CoP–CoM_max-FO, and AP_CoP–CoM_min-FO, indicating their relatively greater contribution to cluster differentiation across both AP and ML control in global and local coordinate systems (Fig 4).

#### Cluster A: efficient propulsion and stability.

Cluster A exhibited the highest walking speed, with both greater paretic EBF and PF and lower LBF. The CoM and CoP maintained forward and backward displacements with smaller lateral deviations throughout the stance phase (ML_CoP–CoM_max-FO) without the reversed CoP–CoM trajectory in the late stance phase (AP_CoP–CoM_min-FO). This suggests that foot placement was not excessively lateralized because of lateral body sway [29], thereby maintaining stability during walking.

In the local coordinate system, participants in cluster A exhibited hindfoot contact and transitioned loading to the medial forefoot during push-off, suggesting the proper function of the three-rocker mechanism. This finding supports the results of previous studies that reported that anterior displacement of CoP is an important indicator of walking ability [12,15,34,35].

#### Cluster B: reduced propulsion with impaired weight shifting and lateral instability.

Compared with cluster A, cluster B exhibited significantly lower PF and walking speed, with a significantly smaller AP_CoP–CoM_min. The presence of a toe-out posture and lateral shift of the CoP away from the CoM suggest impaired lateral control, resulting in more challenging weight shifting to the paretic limb. In individuals with slower walking speeds, the paretic foot tends to be positioned more laterally relative to the pelvis, thus reducing posterior support and limiting adequate forward placement of the nonparetic foot [10,11]. Although excessive braking force during early stance is known to hinder forward progression of the CoM [19], previous studies have also shown that early braking force tends to increase with faster gait speed [8]. In our study, peak EBF and AP_CoP–CoM_max were not significantly different between clusters A and B, despite cluster B exhibiting a slower gait speed. One possible explanation is that hindfoot_CoP_duration in cluster B was significantly shorter than in cluster A, indicating a more anterior CoP position at initial contact, which may have resulted in a comparable EBF. However, the reduced gait speed in cluster B suggested a lower CoM velocity at initial contact, which may lead to insufficient forward progression of the CoM during stance caused by the large EBF, thus contributing to the smaller AP_CoP–CoM_min. Furthermore, increased ML_CoP–CoM_max-FO, ML_CoP–CoM_max, and FPA in the global coordinate system as well as difficulties in hindfoot loading in the local coordinate system suggest that lateral CoP shifting at foot-off may restrict body weight transfer onto the paretic limb. Notably, cluster B also demonstrated significantly more severe motor impairment and included a higher proportion of walking aid users compared with cluster A (Table 2). These clinical factors may underlie the impaired lateral weight shifting and reduced push-off capacity observed in this group.

In contrast to the findings of a previous study [28], no relationship between decreased propulsion and increased LBFs was observed in our study. This could be a compensatory strategy in which lateral foot placement relative to the CoM and increased FPA facilitate toe clearance. Because the LBF is associated with circumduction, changes in lateral foot displacement from the foot to the swing phase may account for this variation [20].

#### Clusters C and D: reduced propulsion and increased late braking force with the CoM and CoP moving closer during late stance.

Cluster D exhibited lower EBF and PF than those in cluster A. Although the reductions in EBF and PF in cluster C were not significant, a trend similar to that in cluster D was observed. This reduction may be partly explained by restricted AP foot placement, as indicated by significantly smaller AP_CoP–CoM_max and AP_CoP–CoM_min than in cluster A. However, in the local coordinate system, cluster C exhibited differing patterns, including a prolonged hindfoot loading duration, shortened forefoot loading duration, and medial CoP shift at foot-off. In individuals with ankle arthrodesis, an increased contact duration in the hindfoot may lead to insufficient forefoot loading, particularly at the first toe, resulting in reduced forefoot loading pressure and impaired push-off during gait [36]. Inappropriate activation of the tibialis anterior during stance in individuals with hemiparesis [28] may reduce the propulsion force by inducing prolonged hindfoot loading and shortened forefoot loading. Furthermore, inadequate push-off caused by sustained ankle dorsiflexion impairs the vertical acceleration of the heel during late stance, contributing to stiff knee gait [37], which may lead to LBF generation [16]. Because of the small sample size of cluster C (n = 4), interpretations of its findings are exploratory. However, cluster D exhibited significantly higher ML_CoP–CoM_max-FO, ML_CoP–CoM_max, and ML_CoP_FO values compared with cluster A. These findings suggest that lateral deviation of the CoP in both the global and local coordinate systems is potentially influenced by inappropriate activation of ankle plantar flexion with inversion observed with central nervous system disorders, which may indicate lateral instability. Consistently, the proportion of walking aid users was significantly higher in cluster D than in cluster A (Table 2), suggesting compensatory strategies for impaired lateral stability. This finding supports the results of a previous study that reported increased lateral displacement of the CoM throughout the gait cycle in response to spasticity-related instability in individuals with hemiparesis [33]. Impairment of the ankle plantar flexors reduces toe clearance, thus leading to compensatory movements such as hip hike and circumduction [20], which also likely contribute to LBF generation [16].

These findings highlight how different CoP–CoM dynamics are associated with reduced PF and LBF generation during hemiparetic gait. Additionally, local CoP-based parameters, such as a prolonged hindfoot loading duration and large lateral CoP shift, provide complementary insight into ankle dysfunction.

### Clinical implications

The stance-phase–derived gait characteristics explored in this study highlight clinically relevant issues. Relationships between the gait metrics and GRFs within each cluster are shown in S4 Fig. In cluster A (positive ML_CoP_FO values), a greater medial shift of the CoP after anterior displacement was positively correlated with a larger LBF; however, in cluster D (negative ML_CoP_FO values), this correlation was negative, suggesting that an excessive medial shift of the CoP represents foot dragging during the load transition from the paretic to the nonparetic limb. This interpretation is supported by the observed relationship between large anterior and medial shifts of the CoP and increased LBF in cluster A. Thus, controlling the CoP position at foot-off may be a relevant target for gait training. Furthermore, targeting the hindfoot loading duration may enhance the PF based on the inverted pendulum mechanism. In cluster B, which had a small AP_CoP_FO, greater anterior displacement of the CoP was correlated with increased PF, emphasizing the importance of decreasing ML_CoP–CoM_max-FO. From a broader perspective, EBF, PF, and LBF are associated with the metrics of the CoP–CoM dynamics. However, a detailed analysis of the CoP metrics could provide important insights into potential stance-phase strategies within each cluster.

**Fig 5:**
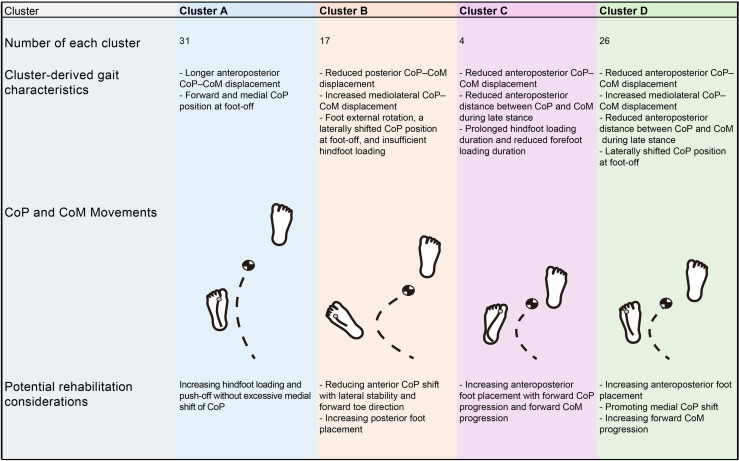
Clinical implications of cluster-derived gait characteristics. Gait characteristics related to each cluster **(A, B, C,** and **D**) and their clinical implications are illustrated. Each cluster shows features related to the center of pressure (CoP)–center of mass (CoM) displacement, mediolateral control, foot placement, and stance phase duration. Illustrations of the CoP and CoM movement sections depict simplified representative gait subgroups for each cluster. These illustrations are schematic and conceptual in nature; therefore, they are intended for illustrative purposes only and were not derived from individual empirical gait trajectories. The table summarizes the key gait characteristics and potential considerations for rehabilitation and physiotherapy for each group.

The potential rehabilitation considerations of each cluster, including propulsion, LBFs, and lateral control strategies, are summarized in Fig 5. Interventions such as robot-assisted gait training to improve heel contact and the AP impulse of GRF in cluster A [38], functional electrical stimulation to correct toe orientation during gait [3], ankle–foot orthoses to enhance support on the paretic limb in cluster B [22], reducing hindfoot impulse in cluster C [39], enhancing ML ankle stability, and preventing foot dragging in cluster D [40] could be considered as potential strategies for addressing heterogeneous gait characteristics.

These findings highlight the importance of categorizing the heterogeneity of hemiparetic gait based on multiple stance-phase gait components. Notably, both the CoP–CoM relationship metrics and CoP-based metrics provided complementary insights into gait dynamics. For instance, the same CoP-based features, such as the anterior and ML shifts of the CoP, had cluster-derived implications for GRFs, including PFs and LBFs. Therefore, such features should be interpreted within the broader context of gait. Similarly, CoP–CoM relationship metrics revealed global postural control characteristics, which were associated with differences in stance-phase gait characteristics across candidate subgroups. Collectively, these results support the clinical utility of combining both CoP-based and CoP–CoM dynamics when evaluating hemiparetic gait. Such an approach may support clinical interpretations of heterogeneous gait impairments and inform future research of targeted rehabilitation.

### Study limitations

Although cluster stability was assessed using ARI-based resampling (1,000 initializations) and the optimal number of clusters was supported by silhouette analysis, elbow method, and PCA-based visualization, a major limitation remains the lack of external validation using independent datasets. Furthermore, although Cluster C showed consistent membership across k = 4 and k = 5 solutions, it comprised only four participants, which substantially limits statistical power for pairwise comparisons. Findings related to Cluster C should therefore be interpreted with caution and regarded as exploratory.

The present study did not include a healthy control cohort; therefore, the observed CoP–CoM parameter values could not be directly compared with normative data. Establishing reference values for these stance-phase–derived parameters in healthy individuals is an important direction for future research.

Additionally, the present analysis did not include a detailed examination of kinematic parameters associated with LBFs. Consequently, the extent to which compensatory mechanisms, such as circumduction and hip hike, occur is unclear. These dynamic changes influence alterations in kinematics [16,20]; therefore, the results of this study suggest that the trajectory characteristics of the CoM and CoP during late stance may reflect these alterations.

The trajectory of the CoM during the second double stance was influenced by the nonparetic limb. Analyses of CoP dynamics in acute stroke have indicated that delayed paretic heel-off or early nonparetic heel-on significantly affects functional outcomes, thus highlighting the importance of CoP dynamics during the second double stance [35]. Additionally, although the decelerated forward progression of CoM, which is one of the factors associated with CoP and CoM convergence, may not be solely influenced by the paretic limb. AP_CoP–CoM_min-FO on the paretic side was significantly correlated with LBFs. These findings suggest that the interaction between the CoM alteration and the CoP position during the paretic late stance phase influences changes in the dynamics of the paretic limb.

Wong et al. detailed the relationship between the AP loading position of the foot, its initiation point, the severity of motor impairment, and GRFs [15], and reduced forward displacement of the CoP during late stance has also been associated with LBF generation [16]. In the present study, we analyzed the characteristics of loading positions in detail to explore candidate biomechanical subgroups. The results suggested that the ML shift of the CoP during push-off, rather than the loading duration of the CoP from the hindfoot to the forefoot, may characterize stance-phase gait characteristics. However, even if the CoP showed a medial shift, as observed in cluster C, the reduction in the forefoot CoP duration suggested that it may not contribute effectively to PF. Therefore, further investigations are required to elucidate the detailed kinematic mechanisms.

Finally, the use of a cane during gait analysis, although limited to the minimum extent necessary to prevent falls, may have influenced ML control of the CoM and should be considered when interpreting the CoP–CoM dynamics observed in this study.

## Conclusions

This comprehensive analysis of clinically relevant gait measures of poststroke hemiparetic gait focused on the stance-phase–derived features of the CoP and CoM dynamics in the transverse plane. Specifically, the relative positions of the CoP and CoM during late stance and local CoP features were closely correlated with PFs and LBFs. Furthermore, clustering analysis of 78 individuals with hemiparetic gait indicated four candidate subgroups with heterogeneous stance-phase CoP–CoM characteristics. These findings provide insights into biomechanical features associated with PF and LBF and offer a descriptive framework for understanding diverse gait impairments. Future studies that incorporate external validation and detailed kinematic analyses may further elucidate compensatory mechanisms, including contributions from the nonparetic limb, and may support the development of personalized gait rehabilitation approaches.

## Supporting information

S1 Fig Relationship between center of pressure and center of mass dynamics and ground reaction forces in individuals with hemiparetic gait. Values represent Spearman’s correlation coefficients. In the correlation heatmaps, colored cells indicate statistically significant correlations (p < 0.05), whereas uncolored cells indicate nonsignificant correlations (p ≥ 0.05).
(TIF)

S2 Fig Cluster selection and stability assessment for k-means clustering. (A) Silhouette coefficient plot for k = 2–10. The score peaked at k = 4, supporting its selection as the optimal number of clusters. (B) Elbow method plot illustrating the sum of squared errors for k = 2–10. No clear inflection point was observed. (C) Histograms of Adjusted Rand Index (ARI) values from 100 repeated k-means runs for k = 3, 4, and 5, reflecting cluster stability across initializations. (D) Cross-tabulation of cluster membership between the k = 4 and k = 5 solutions.
(TIF)

S3 Fig Anteroposterior ground reaction forces during the stance phase in each cluster. The solid line and shaded area indicate the mean and 95% confidence interval in each cluster, respectively.
(TIF)

S4 Fig Relationships between center of pressure and center of mass dynamics and ground reaction forces in each cluster. Each panel indicates a separate cluster. The values represent Spearman’s correlation coefficients. The significance threshold is consistent with that in S1 Fig (p < 0.05; colored cells indicate significant correlations).
(TIF)

S1 Table Differences in gait parameters among clusters (k = 4). Values are expressed as medians (25th, 75th percentiles). P values were obtained using the Kruskal–Wallis test, and effect sizes were calculated using epsilon-squared (ε²). Pairwise comparisons between clusters were conducted using the Steel–Dwass test to identify significant differences. *Significant difference in clusters B, C and D compared with cluster A (p < 0.05). †Significant difference in clusters C and D compared with cluster B (p < 0.05). ‡Significant difference in cluster D compared with cluster C (p < 0.05). AP, anteroposterior; BW, body weight; CoM, center of mass; CoP, center of pressure; FO, foot-off; FPA, foot progression angle; max, maximal; min, minimum; ML, mediolateral.
(DOCX)

S2 Table Differences in gait parameters among clusters (k = 5). Values are expressed as medians (25th, 75th percentiles). P values were obtained using the Kruskal–Wallis test, and effect sizes were calculated using epsilon-squared (ε²). Pairwise comparisons between clusters were conducted using the Steel–Dwass test to identify significant differences. *Significant difference in clusters A’, B, C and D compared with cluster A (p < 0.05). †Significant difference in clusters B, C and D compared with cluster A’ (p < 0.05). ‡Significant difference in cluster C and D compared with cluster B (p < 0.05). §Significant difference in cluster D compared with cluster C (p < 0.05). AP, anteroposterior; BW, body weight; CoM, center of mass; CoP, center of pressure; FO, foot-off; FPA, foot progression angle; max, maximal; min, minimum; ML, mediolateral.
(DOCX)

S1 Dataset Dataset used for analysis. This table provides the anonymized dataset used in this study, including processed gait metrics, ground reaction force variables, cluster assignments, and demographic summaries. The dataset is provided in CSV format.
(CSV)

## References

[1] De Quervain IA, Simon SR, Leurgans S, Pease WS, McAllister D. Gait pattern in the early recovery period after stroke. J Bone Joint Surg Am. 1996;78(10):1506–14. doi: 10.2106/00004623-199610000-00008 8876578

[2] Jørgensen HS, Nakayama H, Raaschou HO, Olsen TS. Recovery of walking function in stroke patients: the Copenhagen Stroke Study. Arch Phys Med Rehabil. 1995;76(1):27–32. doi: 10.1016/s0003-9993(95)80038-7 7811170

[3] Mao YR, Zhao JL, Bian MJ, Lo WLA, Leng Y, Bian RH, et al. Spatiotemporal, kinematic and kinetic assessment of the effects of a foot drop stimulator for home-based rehabilitation of patients with chronic stroke: a randomized clinical trial. J Neuroeng Rehabil. 2022;19(1):56. doi: 10.1186/s12984-022-01036-0 35672756 PMC9172181

[4] Bowden MG, Balasubramanian CK, Neptune RR, Kautz SA. Anterior-posterior ground reaction forces as a measure of paretic leg contribution in hemiparetic walking. Stroke. 2006;37(3):872–6. doi: 10.1161/01.STR.0000204063.75779.8d 16456121

[5] Roerdink M, Beek PJ. Understanding inconsistent step-length asymmetries across hemiplegic stroke patients: impairments and compensatory gait. Neurorehabil Neural Repair. 2011;25(3):253–8. doi: 10.1177/1545968310380687 21041500

[6] Lin P-Y, Yang Y-R, Cheng S-J, Wang R-Y. The relation between ankle impairments and gait velocity and symmetry in people with stroke. Arch Phys Med Rehabil. 2006;87(4):562–8. doi: 10.1016/j.apmr.2005.12.042 16571398

[7] Awad LN, Lewek MD, Kesar TM, Franz JR, Bowden MG. These legs were made for propulsion: advancing the diagnosis and treatment of post-stroke propulsion deficits. J Neuroeng Rehabil. 2020;17(1):139. doi: 10.1186/s12984-020-00747-6 33087137 PMC7579929

[8] Prible D, Fey NP, Yuan Hsiao H. Biomechanical mechanism of peak braking force modulation during increased walking speed in healthy young adults. J Biomech. 2022;144:111311. doi: 10.1016/j.jbiomech.2022.111311 36154983

[9] Lewek MD, Sawicki GS. Trailing limb angle is a surrogate for propulsive limb forces during walking post-stroke. Clin Biomech (Bristol). 2019;67:115–8. doi: 10.1016/j.clinbiomech.2019.05.011 31102839 PMC6635006

[10] Balasubramanian CK, Neptune RR, Kautz SA. Foot placement in a body reference frame during walking and its relationship to hemiparetic walking performance. Clin Biomech (Bristol). 2010;25(5):483–90. doi: 10.1016/j.clinbiomech.2010.02.003 20193972 PMC2881577

[11] Stimpson KH, Heitkamp LN, Embry AE, Dean JC. Post-stroke deficits in the step-by-step control of paretic step width. Gait Posture. 2019;70:136–40. doi: 10.1016/j.gaitpost.2019.03.003 30856525 PMC6474800

[12] Choi H, Kim W-S. Anterior-posterior displacement of center of pressure measured by insole foot pressure measurement system in subacute recovery stage of post-stroke hemiplegia. Technol Health Care. 2018;26(4):649–57. doi: 10.3233/THC-181310 30124457

[13] Kimoto M, Okada K, Mitobe K, Saito M, Kawanobe U, Sakamoto H. Analysis of center of mass and center of pressure displacement in the transverse plane during gait termination in children with cerebral palsy. Gait Posture. 2021;90:106–11. doi: 10.1016/j.gaitpost.2021.07.015 34438291

[14] Van Criekinge T, Saeys W, Hallemans A, Velghe S, Viskens P-J, Vereeck L, et al. Trunk biomechanics during hemiplegic gait after stroke: a systematic review. Gait Posture. 2017;54:133–43. doi: 10.1016/j.gaitpost.2017.03.004 28288334

[15] Wong AM, Pei Y-C, Hong W-H, Chung C-Y, Lau Y-C, Chen CP. Foot contact pattern analysis in hemiplegic stroke patients: an implication for neurologic status determination. Arch Phys Med Rehabil. 2004;85(10):1625–30. doi: 10.1016/j.apmr.2003.11.039 15468022

[16] Ohta M, Tanabe S, Katsuhira J, Tamari M. Kinetic and kinematic parameters associated with late braking force and effects on gait performance of stroke patients. Sci Rep. 2023;13(1):7729. doi: 10.1038/s41598-023-34904-3 37173403 PMC10182027

[17] van Leeuwen AM, van Dieën JH, Daffertshofer A, Bruijn SM. Ankle muscles drive mediolateral center of pressure control to ensure stable steady state gait. Sci Rep. 2021;11(1):21481. doi: 10.1038/s41598-021-00463-8 34728667 PMC8563802

[18] Ogura A, Chujo Y, Mano N, Mori K, Konishi T, Kuwabara T, et al. Effects of ankle joint degree of freedom of knee-ankle-foot orthoses on loading patterns and triceps surae muscle activity on the paretic side in individuals with subacute severe hemiplegia: a retrospective study. J Neuroeng Rehabil. 2024;21(1):150. doi: 10.1186/s12984-024-01432-8 39227980 PMC11373454

[19] Duclos NC, Duclos C, Nadeau S. Slow and faster post-stroke walkers have a different trunk progression and braking impulse during gait. Gait Posture. 2019;68:483–7. doi: 10.1016/j.gaitpost.2018.12.037 30616177

[20] Dean JC, Bowden MG, Kelly AL, Kautz SA. Altered post-stroke propulsion is related to paretic swing phase kinematics. Clin Biomech (Bristol). 2020;72:24–30. doi: 10.1016/j.clinbiomech.2019.11.024 31809919 PMC7089813

[21] Mulroy S, Gronley J, Weiss W, Newsam C, Perry J. Use of cluster analysis for gait pattern classification of patients in the early and late recovery phases following stroke. Gait Posture. 2003;18(1):114–25. doi: 10.1016/s0966-6362(02)00165-0 12855307

[22] Kim H, Kim Y-H, Kim S-J, Choi M-T. Pathological gait clustering in post-stroke patients using motion capture data. Gait Posture. 2022;94:210–6. doi: 10.1016/j.gaitpost.2022.03.007 35367849

[23] Mizuta N, Hasui N, Kai T, Inui Y, Sato M, Ohnishi S, et al. Characteristics of limb kinematics in the gait disorders of post-stroke patients. Sci Rep. 2024;14(1):3082. doi: 10.1038/s41598-024-53616-w 38321081 PMC10847092

[24] Hummel J, Schwenk M, Seebacher D, Barzyk P, Liepert J, Stein M. Clustering approaches for gait analysis within neurological disorders: a narrative review. Digit Biomark. 2024;8(1):93–101. doi: 10.1159/000538270 38721018 PMC11078540

[25] Bowden MG, Clark DJ, Kautz SA. Evaluation of abnormal synergy patterns poststroke: relationship of the Fugl-Meyer Assessment to hemiparetic locomotion. Neurorehabil Neural Repair. 2010;24(4):328–37. doi: 10.1177/1545968309343215 19794132 PMC4434590

[26] Chujo Y, Mori K, Wakida M, Mano N, Kuwabara T, Tanaka H, et al. Diverse plantarflexor module characteristics influence immediate effects of plastic ankle-foot orthosis on gait performance in patients with stroke: a cross-sectional study. Arch Phys Med Rehabil. 2024;105(7):1322–9. doi: 10.1016/j.apmr.2024.02.734 38458374

[27] Yamaguchi T, Suzuki A, Hokkirigawa K. Required coefficient of friction in the anteroposterior and mediolateral direction during turning at different walking speeds. PLoS One. 2017;12(6):e0179817. doi: 10.1371/journal.pone.0179817 28640853 PMC5480978

[28] Turns LJ, Neptune RR, Kautz SA. Relationships between muscle activity and anteroposterior ground reaction forces in hemiparetic walking. Arch Phys Med Rehabil. 2007;88(9):1127–35. doi: 10.1016/j.apmr.2007.05.027 17826457 PMC2367107

[29] Rebula JR, Ojeda LV, Adamczyk PG, Kuo AD. The stabilizing properties of foot yaw in human walking. J Biomech. 2017;53:1–8. doi: 10.1016/j.jbiomech.2016.11.059 28161109 PMC6311129

[30] Werner C, Gönel M, Lerch I, Curt A, Demkó L. Data-driven characterization of walking after a spinal cord injury using inertial sensors. J Neuroeng Rehabil. 2023;20(1):55. doi: 10.1186/s12984-023-01178-9 37120519 PMC10149024

[31] Kettlety SA, Finley JM, Reisman DS, Schweighofer N, Leech KA. Speed-dependent biomechanical changes vary across individual gait metrics post-stroke relative to neurotypical adults. J Neuroeng Rehabil. 2023;20(1):14. doi: 10.1186/s12984-023-01139-2 36703214 PMC9881336

[32] Hubert L, Arabie P. Comparing partitions. J Classif. 1985;2:193–218. doi: 10.1007/BF01908075

[33] De Bujanda E, Nadeau S, Bourbonnais D. Pelvic and shoulder movements in the frontal plane during treadmill walking in adults with stroke. J Stroke Cerebrovasc Dis. 2004;13(2):58–69. doi: 10.1016/j.jstrokecerebrovasdis.2004.02.006 17903951

[34] Chisholm AE, Perry SD, McIlroy WE. Inter-limb centre of pressure symmetry during gait among stroke survivors. Gait Posture. 2011;33(2):238–43. doi: 10.1016/j.gaitpost.2010.11.012 21167716

[35] Jeon E-T, Lee S-H, Eun M-Y, Jung J-M. Center of pressure- and machine learning-based gait score and clinical risk factors for predicting functional outcome in acute ischemic stroke. Arch Phys Med Rehabil. 2024;105(12):2277–85. doi: 10.1016/j.apmr.2024.08.006 39187003

[36] Chopra S, Crevoisier X. Bilateral gait asymmetry associated with tibiotalocalcaneal arthrodesis versus ankle arthrodesis. Foot Ankle Surg. 2021;27(3):332–8. doi: 10.1016/j.fas.2020.12.006 33358603

[37] Campanini I, Merlo A, Damiano B. A method to differentiate the causes of stiff-knee gait in stroke patients. Gait Posture. 2013;38(2):165–9. doi: 10.1016/j.gaitpost.2013.05.003 23755883

[38] Forrester LW, Roy A, Hafer-Macko C, Krebs HI, Macko RF. Task-specific ankle robotics gait training after stroke: a randomized pilot study. J Neuroeng Rehabil. 2016;13(1):51. doi: 10.1186/s12984-016-0158-1 27255156 PMC4890526

[39] Nolan KJ, Yarossi M. Weight transfer analysis in adults with hemiplegia using ankle foot orthosis. Prosthet Orthot Int. 2011;35(1):45–53. doi: 10.1177/0309364610393061 21515889

[40] Choo YJ, Chang MC. Effectiveness of an ankle-foot orthosis on walking in patients with stroke: a systematic review and meta-analysis. Sci Rep. 2021;11(1):15879. doi: 10.1038/s41598-021-95449-x 34354172 PMC8342539

