# Supplementary figures and images for: Data-driven analysis of heterogeneous gait subgroups and ground reaction forces based on integrated center of pressure–center of mass dynamics in poststroke hemiparesis
Source: PLoS One. 2026 Jul 20;21(7):e0354290. doi: 10.1371/journal.pone.0354290 (PMC13384309; doi:10.1371/journal.pone.0354290)

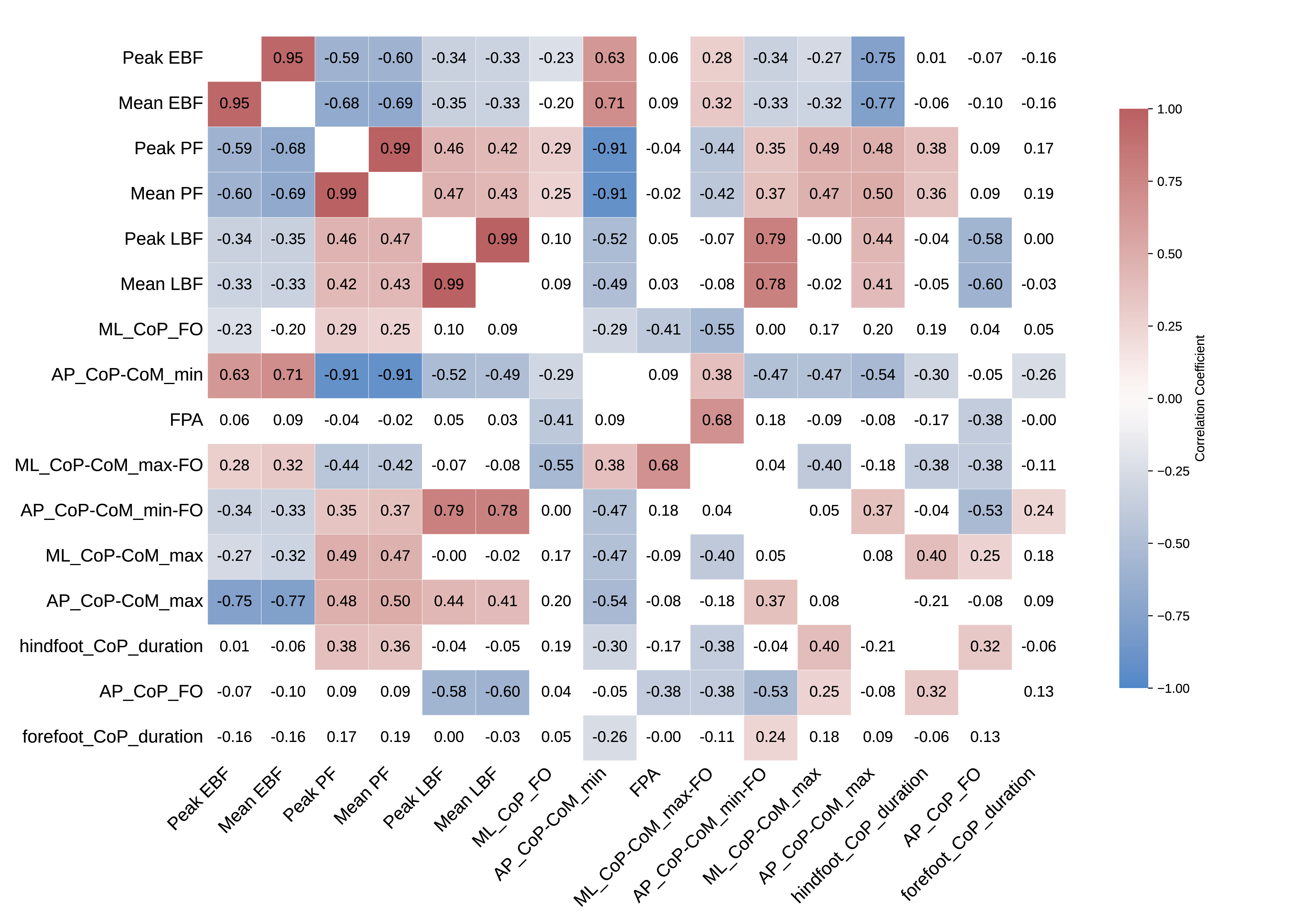

Supplement: S1 Fig — Values represent Spearman’s correlation coefficients. In the correlation heatmaps, colored cells indicate statistically significant correlations (p < 0.05), whereas uncolored cells indicate nonsignificant correlations (p ≥ 0.05). (TIF) [file pone.0354290.s001.tif]

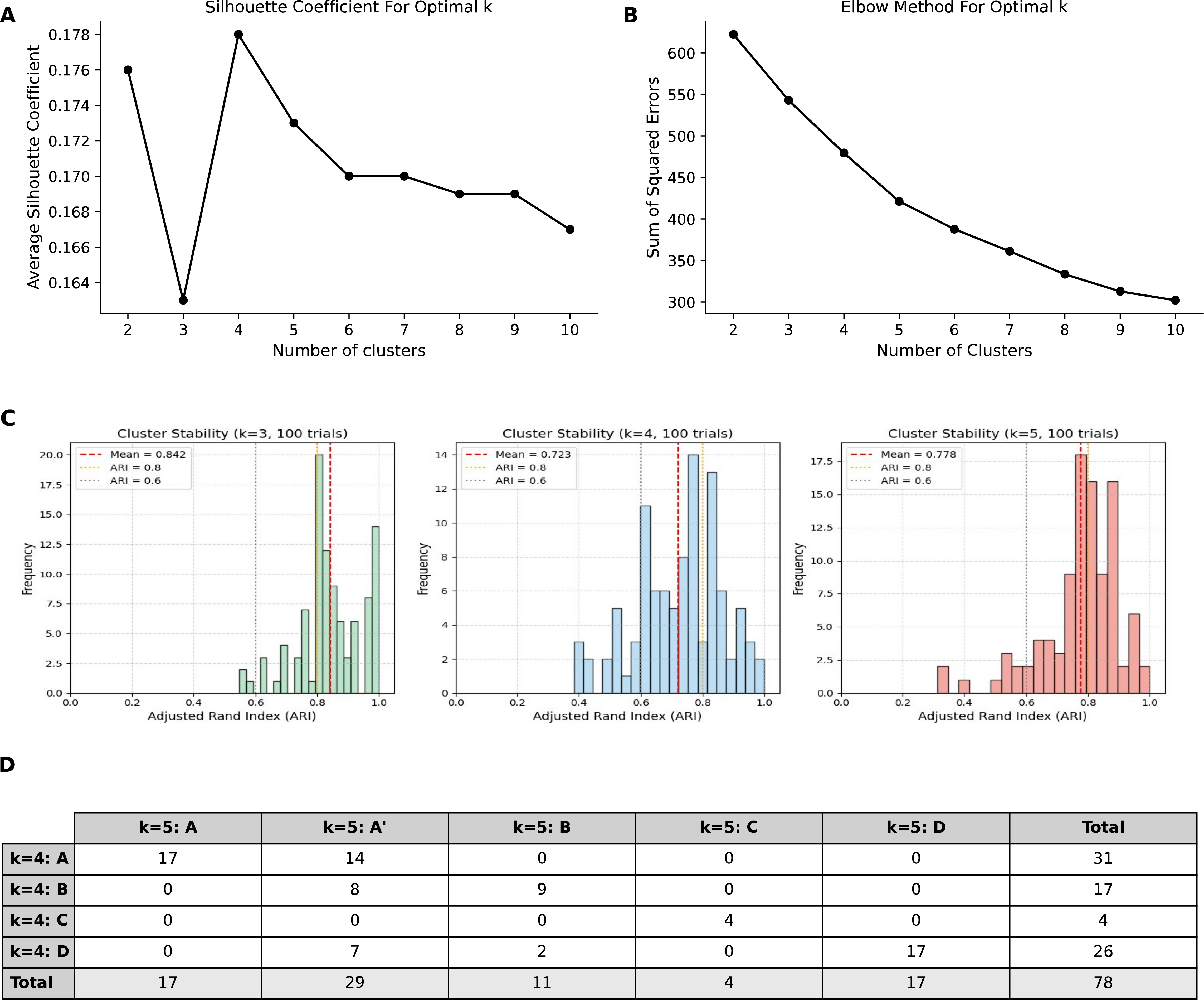

Supplement: S2 Fig — (A) Silhouette coefficient plot for k = 2–10. The score peaked at k = 4, supporting its selection as the optimal number of clusters. (B) Elbow method plot illustrating the sum of squared errors for k = 2–10. No clear inflection point was observed. (C) Histograms of Adjusted Rand Index (ARI) values from 100 repeated k-means runs for k = 3, 4, and 5, reflecting cluster stability across initializations. (D) Cross-tabulation of cluster membership between the k = 4 and k = 5 solutions. (TIF) [file pone.0354290.s002.tif]

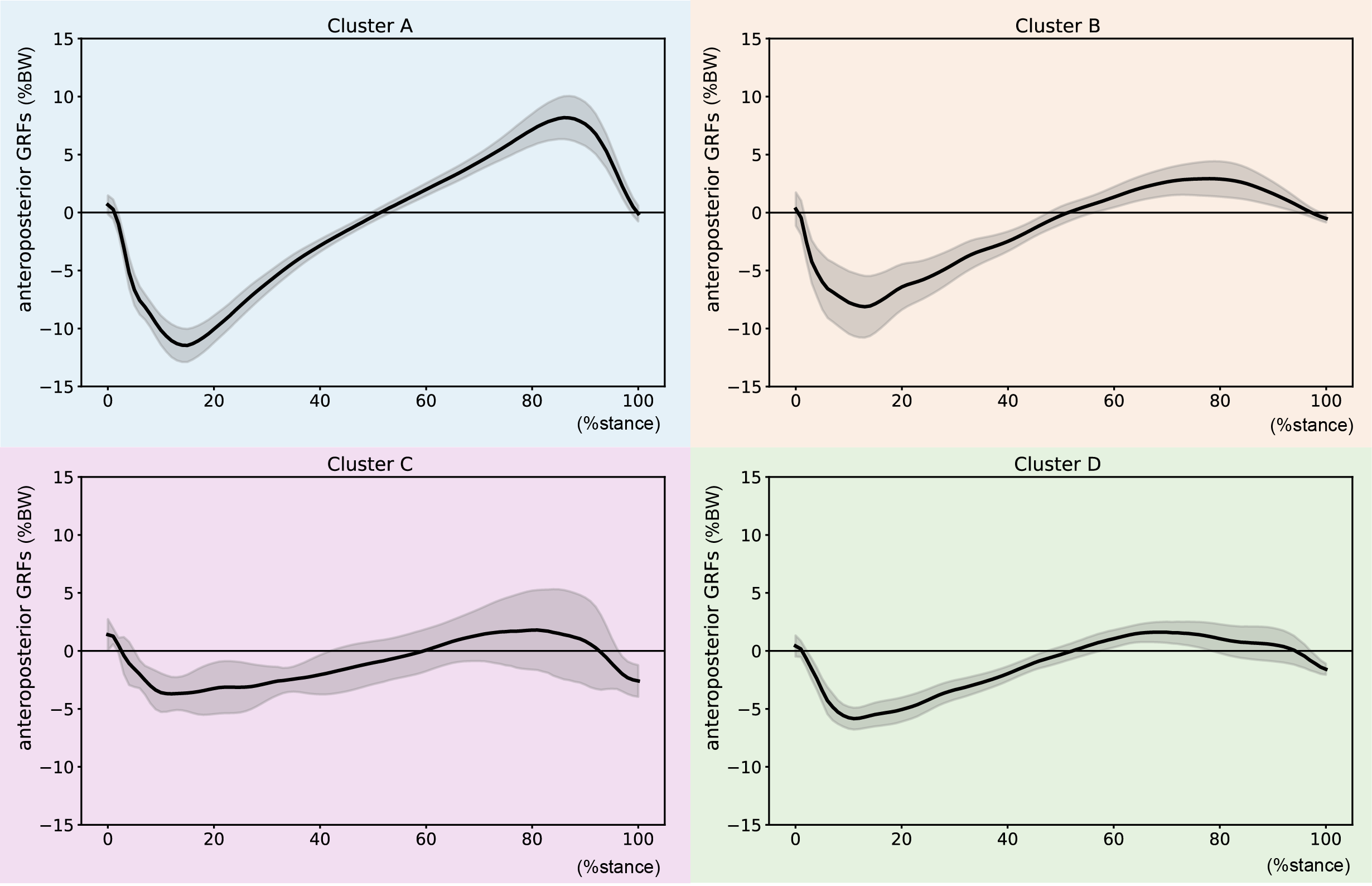

Supplement: S3 Fig — The solid line and shaded area indicate the mean and 95% confidence interval in each cluster, respectively. (TIF) [file pone.0354290.s003.tif]

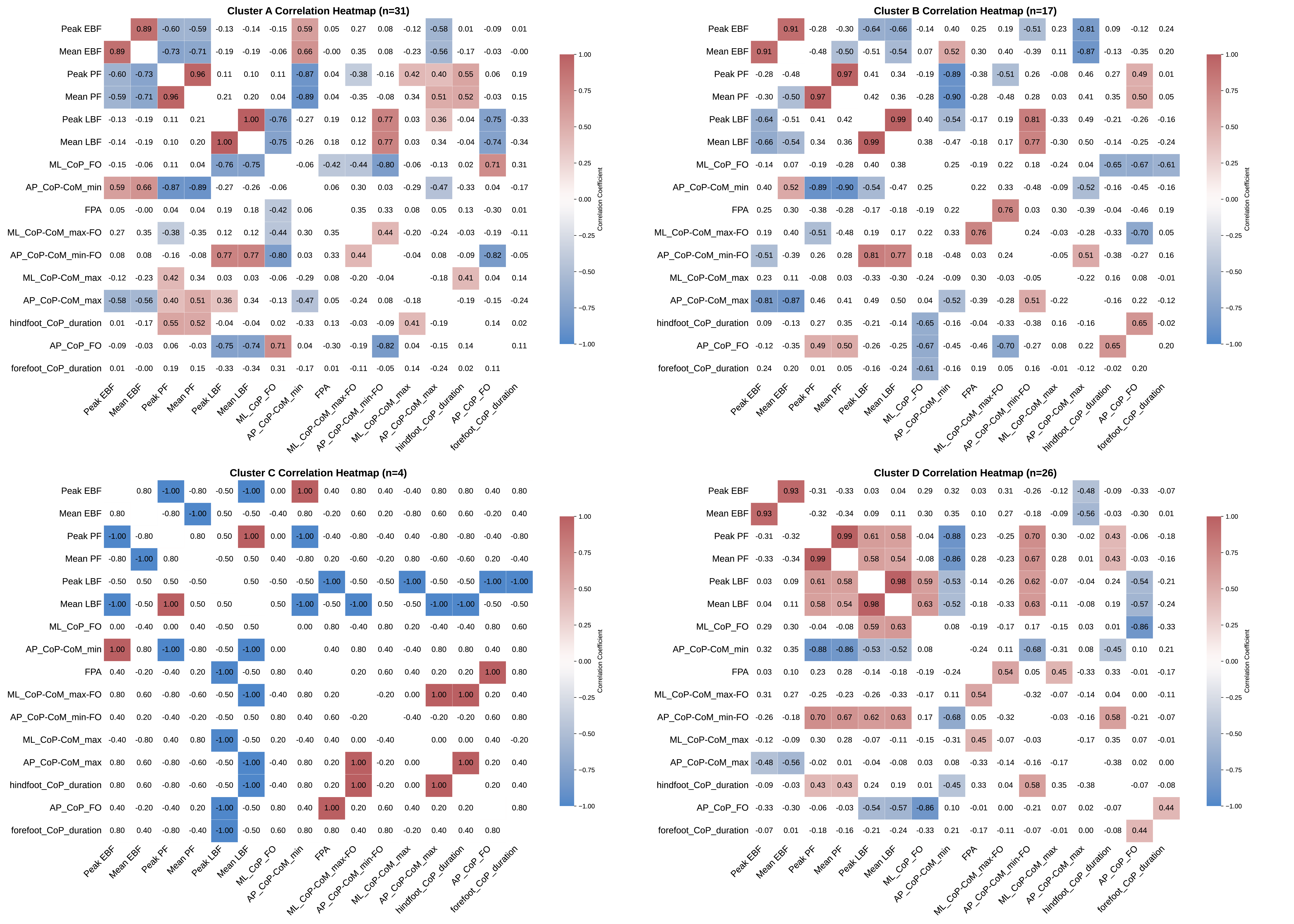

Supplement: S4 Fig — Each panel indicates a separate cluster. The values represent Spearman’s correlation coefficients. The significance threshold is consistent with that in S1 Fig (p < 0.05; colored cells indicate significant correlations). (TIF) [file pone.0354290.s004.tif]
