# Supplementary material for: Data-driven analysis of heterogeneous gait subgroups and ground reaction forces based on integrated center of pressure–center of mass dynamics in poststroke hemiparesis
Source: PLoS One. 2026 Jul 20;21(7):e0354290. doi: 10.1371/journal.pone.0354290 (PMC13384309; doi:10.1371/journal.pone.0354290)
Supplement: S1 Table — Values are expressed as medians (25th, 75th percentiles). P values were obtained using the Kruskal–Wallis test, and effect sizes were calculated using epsilon-squared (ε²). Pairwise comparisons between clusters were conducted using the Steel–Dwass test to identify significant differences. *Significant difference in clusters B, C and D compared with cluster A (p < 0.05). †Significant difference in clusters C and D compared with cluster B (p < 0.05). ‡Significant difference in cluster D compared with cluster C (p < 0.05). AP, anteroposterior; BW, body weight; CoM, center of mass; CoP, center of pressure; FO, foot-off; FPA, foot progression angle; max, maximal; min, minimum; ML, mediolateral. (DOCX) [file pone.0354290.s005.docx]

| **Gait parameters** | **Cluster A** | **Cluster B** | **Cluster C** | **Cluster D** | ***P* (ε²)** | **Mean decrease accuracy** |
| --- | --- | --- | --- | --- | --- | --- |
| ML_CoP_FO (cm/height) | 4.1 (-3.2, 6.1) | -4.2 (-4.9, -3.1) * | 6.3 (5.6, 7.3) † | -5.3 (-6.2, -3.6) *‡ | <0.001 (0.43) | 15.9 |
| AP_CoP–CoM_min (cm/height) | -12.7 (-15.8, -10.4) | -8.8 (-10.7, -5.9) * | -5.3 (-7.8, -3.3) * | -6.0 (-8.1, -3.2) * | <0.001 (0.47) | 15.6 |
| FPA (deg) | 7.9 (5.1, 11.7) | 28.1 (20.2, 31.7) * | 4.4 (1.8, 6.5) † | 10.7 (3.3, 16.6) † | <0.001 (0.37) | 14.3 |
| ML_CoP–CoM_max-FO (cm/height) | 1.9 (1.4, 3.0) | 5.7 (4.5, 7.2) * | 1.5 (1.3, 1.7) † | 3.7 (2.8, 4.5) *†‡ | <0.001 (0.54) | 13.6 |
| AP_CoP–CoM_min-FO (cm/height) | -0.8 (-1.5, 0.0) | -0.7 (-1.1, -0.2) | -3.6 (-4.0, -2.9) *† | -2.3 (-3.2, -1.2) *† | <0.001 (0.28) | 10.0 |
| ML_CoP–CoM_max (cm/height) | -4.6 (-5.5, -4.1) | -7.0 (-8.4, -6.3) * | -7.0 (-8.0, -5.7) | -6.0 (-6.8, -5.3) *† | <0.001 (0.31) | 8.5 |
| AP_CoP–CoM_max (cm/height) | 12.6 (11.3, 14.8) | 11.3 (10.4, 14.6) | 8.8 (8.1, 9.4) * | 9.1 (7.4, 11.1) * | <0.001 (0.29) | 7.5 |
| Hindfoot_CoP_duration | 0.03 (0.01, 0.07) | 0 (0, 0.02) * | 0.25 (0.22, 0.26) *† | 0.02 (0.00, 0.05) ‡ | <0.001 (0.25) | 6.9 |
| AP_CoP_FO (cm/height) | 15.6 (14.4, 17.8) | 13.89 (12.9, 15.2) * | 16.7 (16.1, 17.8) † | 16.2 (14.7, 17.1) † | <0.001 (0.21) | 4.9 |
| Forefoot_CoP_duration | 0.32 (0.24, 0.34) | 0.23 (0.14, 0.34) | 0.13 (0.09, 0.17) * | 0.24 (0.13, 0.29) * | 0.007 (0.12) | 2.7 |
| Peak paretic early braking force (%BW) | -12.35 (-16.04, -9.22) | -8.41 (-12.04, -5.09) | -5.30 (-5.79, -4.42) * | -6.41 (-8.03, -5.21) * | <0.001 (0.31) |  |
| Mean paretic early braking force (%BW) | -6.06 (-7.87, -5.41) | -4.08 (-5.99, -2.73) * | -2.96 (-3.61, -2.11) * | -3.42 (-4.63, -2.75) * | <0.001 (0.33) |  |
| Peak paretic propulsion force (%BW) | 9.37 (5.19, 12.20) | 3.90 (1.46, 5.88) * | 2.79 (0.05, 5.56) | 2.10 (1.11, 4.56) * | <0.001 (0.32) |  |
| Mean paretic propulsion force (%BW) | 5.39 (2.99, 6.37) | 2.29 (0.96, 3.19) * | 1.18 (0.04, 2.53) | 1.18 (0.77, 2.60) * | <0.001 (0.31) |  |
| Peak paretic late braking force (%BW) | -0.11 (-1.25, 0) | -0.51 (-1.26, -0.06) | -3.64 (-3.87, -2.18) | -1.76 (-2.60, -1.10) *† | 0.001 (0.17) |  |
| Mean paretic late braking force (%BW) | -0.11 (-0.86, 0) | -0.32 (-0.80, -0.06) | -2.42 (-2.46, -1.39) | -0.94 (-1.60, -0.64) *† | 0.005 (0.15) |  |
| Gait speed (cm/sec) | 82.4 (72.2, 97.6) | 47.3 (30.6, 60.0) * | 45.7 (37.7, 56.3) * | 43.0 (29.3, 56.9) * | <0.001 (0.42) |  |
| Paretic stance duration (%cycle) | 61.3 (58.7, 62.8) | 59.3 (56.7, 65.1) | 60.8 (59.5, 62.6) | 63.2 (59.2, 66.5) | 0.234 (0.02) |  |
| Nonparetic stance duration (%cycle) | 66.2 (63.7, 69.6) | 73.2 (72.1, 76.9) * | 69.2 (65.5, 74.2) | 71.0 (67.4, 77.4) * | <0.001 (0.26) |  |
| Symmetry stance duration | 0.48 (0.46, 0.49) | 0.44 (0.42, 0.47) * | 0.47 (0.46, 0.48) | 0.46 (0.45, 0.49) | 0.002 (0.16) |  |
