# Supplementary material for: Data-driven analysis of heterogeneous gait subgroups and ground reaction forces based on integrated center of pressure–center of mass dynamics in poststroke hemiparesis
Source: PLoS One. 2026 Jul 20;21(7):e0354290. doi: 10.1371/journal.pone.0354290 (PMC13384309; doi:10.1371/journal.pone.0354290)
Supplement: S2 Table — Values are expressed as medians (25th, 75th percentiles). P values were obtained using the Kruskal–Wallis test, and effect sizes were calculated using epsilon-squared (ε²). Pairwise comparisons between clusters were conducted using the Steel–Dwass test to identify significant differences. *Significant difference in clusters A’, B, C and D compared with cluster A (p < 0.05). †Significant difference in clusters B, C and D compared with cluster A’ (p < 0.05). ‡Significant difference in cluster C and D compared with cluster B (p < 0.05). §Significant difference in cluster D compared with cluster C (p < 0.05). AP, anteroposterior; BW, body weight; CoM, center of mass; CoP, center of pressure; FO, foot-off; FPA, foot progression angle; max, maximal; min, minimum; ML, mediolateral. (DOCX) [file pone.0354290.s006.docx]

| **Gait parameters** | **Cluster A (n=17)** | **Cluster A' (n=29)** | **Cluster B (n=11)** | **Cluster C (n=4)** | **Cluster D (n=17)** | ***P* (ε²)** | **Mean Decrease Accuracy** |
| --- | --- | --- | --- | --- | --- | --- | --- |
| ML_CoP_FO (cm/height) | 5.7 (4.5, 7.1) | -3.7 (-4.9, -2.6) * | -3.8 (-4.2, -3.2) * | 6.3 (5.6, 7.3) †‡ | -6.1 (-6.6, -4.0) *†‡§ | <0.001 (0.62) | 19.9 |
| AP_CoP–CoM_min-FO (cm/height) | -1.4 (-1.7, -1.0) | -0.2 (-0.8, -0.0) * | -1.1 (-2.2, -0.5) | -3.6 (-4.0, -2.9) † | -2.6 (-3.5, -2.3) *†‡ | <0.001 (0.55) | 19.3 |
| ML_CoP–CoM_max-FO (cm/height) | 1.6 (1.1, 1.8) | 3.1 (2.6, 4.0) * | 6.9 (6.1, 8.0) *† | 1.5 (1.3, 1.7) †‡ | 3.8 (2.5, 4.6) *‡ | <0.001 (0.57) | 15.4 |
| AP_CoP–CoM_min (cm/height) | -12.9 (-16.3, -10.2) | -11.1 (-13.3, -9.3) | -5.9 (-7.1, -3.7) *† | -5.3 (-7.8, -3.3) | -4.0 (-6.8, -2.9) *† | <0.001 (0.55) | 13.0 |
| FPA (deg) | 97.5 (94.2, 99.0) | 104.4 (97.9, 107.6) * | 118.2 (113.6, 122.3) *† | 94.4 (91.8, 96.5) ‡ | 99.3 (93.1, 104.1) ‡ | <0.001 (0.39) | 9.8 |
| AP_CoP_FO (cm/height) | 17.1 (15.0, 17.8) | 14.7 (13.8, 15.6) * | 13.6 (12.4, 13.8) *† | 16.7 (16.1, 17.8) †‡ | 16.9 (15.8, 17.6) †‡ | <0.001 (0.48) | 8.4 |
| Hindfoot_CoP_duration | 0.03 (0.02, 0.06) | 0.02 (0.01, 0.06) | 0.00 (0.00, 0.02) | 0.25 (0.22, 0.26) *†‡ | 0.01 (0.00, 0.04) § | 0.002 (0.18) | 5.8 |
| AP_CoP–CoM_max (cm/Height) | 12.9 (11.8, 15.5) | 11.9 (10.6, 14.6) | 10.4 (6.07, 11.0) *† | 8.8 (8.1, 9.4) | 9.7 (7.6, 11.3) * | <0.001 (0.23) | 4.6 |
| ML_CoP–CoM_max (cm/Height) | -4.8 (-5.7, -3.9) | -5.3 (-6.9, -4.5) | -6.8 (-8.8, -6.2) * | -7.0 (-8.0, -5.6) | -5.9 (-6.7, -5.8) * | 0.004 (0.15) | 2.0 |
| Forefoot_CoP_duration | 0.34 (0.27, 0.40) | 0.27 (0.17, 0.32) | 0.15 (0.10, 0.31) | 0.13 (0.09, 0.17) | 0.27 (0.19, 0.31) | 0.015 (0.12) | 1.8 |
| Peak paretic early braking force (%BW) | -12.67 (-16.70, -10.76) | -9.30 (-13.35, -7.44) | -5.54 (-8.21, -3.78) * | -5.30 (-5.79, -4.42) * | -6.56 (-8.23, -5.91) * | <0.001 (0.31) |  |
| Mean paretic early braking force (%BW) | -6.06 (-8.13, -5.78) | -5.00 (-6.38, -3.52) | -3.24 (-3.81, -2.01) *† | -2.96 (-3.61, -2.11) * | -3.52 (-4.87, -2.68) * | <0.001 (0.32) |  |
| Peak paretic propulsion force (%BW) | 10.63 (6.82, 12.26) | 5.61 (4.31, 9.01) | 1.46 (1.01, 3.78) *† | 2.78 (0.05, 5.56) | 1.70 (0.46, 2.15) *† | <0.001 (0.45) |  |
| Mean paretic propulsion force (%BW) | 5.68 (3.43, 6.50) | 3.19 (2.29, 5.19) | 0.96 (0.38, 2.21) *† | 1.18 (0.04, 2.53) | 1.00 (0.31, 1.34) *† | <0.001 (0.44) |  |
| Peak paretic late braking force (%BW) | -1.20 (-1.80, -0.11) | -0.02 (-0.53, 0.00) * | -1.07 (-1.72, -0.30) | -3.64 (-3.87, -2.18) † | -2.38 (-3.37, -1.79) †‡ | <0.001 (0.40) |  |
| Mean paretic late braking force (%BW) | -0.66 (-1.15, -0.11) | -0.02 (-0.38, 0.00) * | -0.75 (-0.97, -0.21) | -2.42 (-2.46, -1.39) | -1.55 (-2.15, -0.97) †‡ | <0.001 (0.38) |  |
| Gait speed (cm/sec) | 96.9 (73.4, 101.3) | 62.9 (49.7, 82.4) * | 30.6 (17.9, 36.2) *† | 45.7 (37.7, 56.3) * | 40.0 (28.0, 56.4) *† | <0.001 (0.57) |  |
| Paretic stance duration (%cycle) | 59.8 (58.3, 62.0) | 62.6 (58.4, 63.6) | 60.0 (57.3, 67.3) | 60.8 (59.5, 62.6) | 63.1 (59.9, 66.2) | 0.262 (0.02) |  |
| Nonparetic stance duration (%cycle) | 66.2 (63.6, 69.6) | 68.5 (65.4, 73.1) | 74.5 (71.8, 83.8) *† | 69.2 (65.5, 74.2) | 72.7 (69.2, 77.8) * | <0.001 (0.23) |  |
| Symmetry stance duration | 0.47 (0.46, 0.49) | 0.48 (0.46, 0.49) | 0.45 (0.43, 0.46) * | 0.47 (0.46, 0.48) | 0.46 (0.44, 0.47) | 0.035 (0.09) |  |
